# Incidence of End-Stage Renal Disease Attributed to Diabetes Among Persons with Diagnosed Diabetes — United States and Puerto Rico, 2000–2014

**DOI:** 10.15585/mmwr.mm6643a2

**Published:** 2017-11-03

**Authors:** Nilka Rios Burrows, Israel Hora, Linda S. Geiss, Edward W. Gregg, Ann Albright

**Affiliations:** 1 Division of Diabetes Translation, National Center for Chronic Disease Prevention and Health Promotion, CDC.

During 2014, 120,000 persons in the United States and Puerto Rico began treatment for end-stage renal disease (ESRD) (i.e., kidney failure requiring dialysis or transplantation) (*1*). Among these persons, 44% (approximately 53,000 persons) had diabetes listed as the primary cause of ESRD (ESRD-D) (*1*). Although the number of persons initiating ESRD-D treatment each year has increased since 1980 (*1*,*2*), the ESRD-D incidence rate among persons with diagnosed diabetes has declined since the mid-1990s (*2*,*3*). To determine whether ESRD-D incidence has continued to decline in the United States overall and in each state, the District of Columbia (DC), and Puerto Rico, CDC analyzed 2000–2014 data from the U.S. Renal Data System and the Behavioral Risk Factor Surveillance System. During that period, the age-standardized ESRD-D incidence among persons with diagnosed diabetes declined from 260.2 to 173.9 per 100,000 diabetic population (33%), and declined significantly in most states, DC, and Puerto Rico. No state experienced an increase in ESRD-D incidence rates. Continued awareness of risk factors for kidney failure and interventions to improve diabetes care might sustain and improve these trends.

The U.S. Renal Data System collects, analyzes, and distributes ESRD clinical and claims data to the Centers for Medicare & Medicaid Services (CMS) (*1*). In addition to demographic information, the U.S. Renal Data System includes the date patients were first treated and the primary cause of ESRD from the CMS Medical Evidence Report that health care providers are required by law to complete for each new patient with ESRD (*1*). For this analysis, the number of persons aged ≥18 years initiating ESRD treatment (i.e., dialysis or transplantation) who had diabetes listed as the primary cause of ESRD in each state, DC, and Puerto Rico during 2000–2014 were obtained from the U.S. Renal Data System. Throughout the period, 44%–45% of the new ESRD cases were ESRD-D (*1*).

The Behavioral Risk Factor Surveillance System (BRFSS) conducts state-based, random-digit–dialed telephone surveys in the 50 states, DC, and Puerto Rico and other U.S. territories. BRFSS respondents were classified as having diagnosed diabetes if they answered “yes” to the question, “Has a doctor ever told you that you have diabetes?” Women who were told that they had diabetes only during pregnancy were classified as not having diabetes. BRFSS data were weighted to estimate the number of noninstitutionalized persons aged ≥18 years with diagnosed diabetes in each state, DC, and Puerto Rico. In 2011, BRFSS changed sampling and weighting methodology and added cell phone respondents; however, these changes did not appear to affect overall estimates of self-reported diabetes (*4*). In 2014, the median BRFSS response rate for all states and territories was 40.5% (cell phone) and 48.7% (landline).*

ESRD-D incidence was calculated by dividing the number of persons initiating ESRD-D treatment by the estimated number of persons with diagnosed diabetes in each state, DC, and Puerto Rico. Data were analyzed using statistical software to estimate standard errors and calculate 95% confidence intervals (CIs), and were age-standardized by the direct method based on the 2000 U.S. standard population. Joinpoint regression was used for trend analyses (*5*). Joinpoint regression uses permutation tests to determine whether the rate of change for each trend segment is significantly different from zero (p value <0.05) to identify points (i.e., joinpoints) where linear trends change significantly in direction or magnitude (e.g., zero joinpoints indicates a straight line). In the final model, each trend segment is described by an annual percentage change and the trend for the entire study period is described by the average annual percentage change (AAPC), both with 95% CIs. Alaska, Vermont, and Wyoming were excluded from the trend analysis because of the small annual number (<50) of new ESRD-D cases identified during the study period.

During 2000–2014, the total number of adults aged ≥18 years in the United States, DC, and Puerto Rico who began ESRD-D treatment each year increased from 42,236 (state range = 32–5,117) to 53,382 (state range = 47–7,228) (AAPC = 1.5% per year [95% CI = 1.2%–1.8%], p<0.001) (Figure 1). From 2000 to 2014, among 47 states, DC, and Puerto Rico, the age-standardized ESRD-D incidence decreased 33% (AAPC = ‑2.8% per year [95% CI = -3.3% to -2.3%], p<0.001), from 260.2 (state range = 171.2–569.6) to 173.9 (state range = 81.7–363.6) per 100,000 persons with diabetes (Table) (Figure 1). During 2000–2014, rates declined significantly in most states, DC, and Puerto Rico (Table) (Figure 2). In Kansas and Utah, rates declined and then leveled off. From 2000 to 2014, rates did not decline significantly in California, Hawaii, Mississippi, or Montana (Table). In 2000, the rate was ≥217.5 per 100,000 persons with diabetes in 41 states, DC, and Puerto Rico, and the rate was not <164.5 in any state; in 2014, the rate was ≥217.5 in five states and DC, and was <164.5 in 24 states (Table) (Figure 2).

**FIGURE 1:**
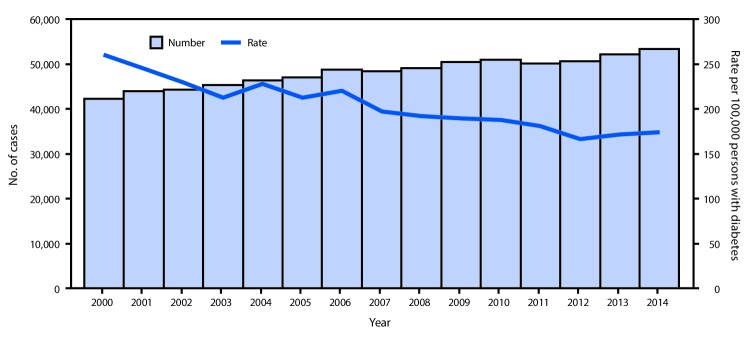
Number and rate* of adults aged ≥18 years who began treatment for end-stage renal disease attributed to diabetes (ESRD-D), U.S. states, the District of Columbia, and Puerto Rico, 2000–2014^†^ * Rate per 100,000 persons with diabetes and age-standardized to the 2000 U.S. standard population, excluding Alaska, Vermont, and Wyoming because of the small annual number (<50) of new ESRD-D cases during the study period. ^†^ In 2011, the Behavioral Risk Factor Surveillance System (BRFSS) survey changed sampling and weighting methodology and added cell phone respondents; however, this change did not appear to affect overall estimates of self-reported diabetes. BRFSS estimates of the population with self-reported diabetes were used to calculate ESRD-D incidence rates.

**TABLE:** Age-standardized incidence* of end-stage renal disease attributed to diabetes (ESRD-D) among adults aged ≥18 years with diagnosed diabetes, by state and territory^†^ — U.S. states, the District of Columbia, and Puerto Rico, 2000–2014

State/Territory	Rate		% Change	Trend analysis	
	2000	2014		AAPC (95% CI)	p value
Alabama	294.7	176.3	-40	-2.7 (-3.8 to -1.5)	<0.001
Arizona	405.7	196.2	-52	-4.3 (-5.7 to -3.0)	<0.001
Arkansas	249.5	155.3	-38	-3.3 (-4.7 to -1.9)	<0.001
California	227.2	188.3	-17	-1.4 (-2.8 to 0.1)	0.06
Colorado	290.5	142.3	-51	-4.5 (-6.1 to -2.9)	<0.001
Connecticut	289.8	131.5	-55	-4.2 (-5.7 to -2.6)	<0.001
Delaware	315.4	135.8	-57	-4.0 (-5.6 to -2.4)	<0.001
District of Columbia	569.6	304.8	-46	-2.9 (-5.2 to -0.5)	0.02
Florida	248.6	142.4	-43	-2.9 (-4.0 to -1.8)	<0.001
Georgia	288.5	166.3	-42	-3.8 (-5.2 to -2.4)	<0.001
Hawaii	557.8	363.6	-35	-1.6 (-3.5 to 0.2)	0.08
Idaho	247.9	166.7	-33	-4.8 (-8.4 to -1.2)	0.01
Illinois	276.8	187.5	-32	-3.0 (-4.4 to -1.6)	<0.001
Indiana	279.7	180.8	-35	-2.3 (-3.5 to -1.2)	<0.001
Iowa	217.4	128.0	-41	-4.7 (-6.7 to -2.7)	<0.001
Kansas^§^	273.3	143.1	-48	-3.7 (-5.1 to -2.3)	<0.001
Kentucky	254.7	143.5	-44	-2.5 (-3.6 to -1.5)	<0.001
Louisiana	337.9	219.8	-35	-4.2 (-5.5 to -2.8)	<0.001
Maine	224.7	114.3	-49	-6.0 (-8.4 to -3.6)	<0.001
Maryland	255.1	160.8	-37	-4.8 (-6.1 to -3.5)	<0.001
Massachusetts	202.9	101.5	-50	-4.9 (-5.8 to -4.0)	<0.001
Michigan	237.2	215.9	-9	-3.1 (-4.2 to -2.0)	<0.001
Minnesota	291.0	123.0	-58	-4.7 (-5.7 to -3.7)	<0.001
Mississippi	287.1	219.3	-24	-1.0 (-2.4 to 0.5)	0.19
Missouri	263.9	150.7	-43	-3.2 (-4.5 to -2.0)	<0.001
Montana	230.0	138.2	-40	-2.2 (-4.5 to 0.2)	0.07
Nebraska	280.5	106.1	-62	-5.4 (-7.2 to -3.5)	<0.001
Nevada	222.1	166.2	-25	-4.1 (-5.6 to -2.5)	<0.001
New Hampshire	350.8	81.7	-77	-4.6 (-7.1 to -2.0)	0.002
New Jersey	292.0	189.6	-35	-2.5 (-3.4 to -1.6)	<0.001
New Mexico	358.2	210.1	-41	-4.5 (-5.8 to -3.2)	<0.001
New York	243.2	155.5	-36	-3.3 (-4.4 to -2.2)	<0.001
North Carolina	304.9	177.3	-42	-3.8 (-4.6 to -2.9)	<0.001
North Dakota	235.6	186.4	-21	-3.0 (-5.0 to -1.0)	0.007
Ohio	299.7	164.4	-45	-3.0 (-4.3 to -1.6)	<0.001
Oklahoma	341.0	190.8	-44	-4.3 (-5.5 to -3.0)	<0.001
Oregon	171.2	148.3	-13	-2.4 (-4.4 to -0.4)	0.02
Pennsylvania	245.6	159.9	-35	-3.0 (-3.9 to -2.0)	<0.001
Rhode Island	176.2	136.8	-22	-4.3 (-7.2 to -1.3)	0.01
South Carolina	298.1	202.7	-32	-3.4 (-5.2 to -1.6)	0.001
South Dakota	265.8	227.1	-15	-3.4 (-4.8 to -1.9)	<0.001
Tennessee	250.5	145.3	-42	-3.0 (-4.1 to -1.9)	<0.001
Texas	342.5	220.9	-36	-2.3 (-3.7 to -0.9)	0.003
Utah^¶^	205.2	156.2	-24	-3.7 (-6.5 to -0.8)	0.01
Virginia	265.7	196.5	-26	-4.3 (-5.9 to -2.7)	<0.001
Washington	176.1	145.0	-18	-1.8 (-2.6 to -0.9)	<0.001
West Virginia	330.6	178.2	-46	-2.7 (-4.4 to -0.9)	0.006
Wisconsin	232.7	174.7	-25	-3.6 (-5.1 to-2.1)	<0.001
**United States**	**260.6**	**173.4**	-**33**	**-2.8 (-3.3 to** -**2.3)**	**<0.001**
Puerto Rico	240.8	207.8	-14	-1.5 (-2.4 to -0.7)	0.002
**Total**	**260.2**	**173.9**	**-33**	**-2.8 (**-**3.3 to** -**2.3)**	**<0.001**

**Abbreviations:** AAPC = average annual percentage change; APC = annual percentage change; CI = confidence interval.

* Rate per 100,000 persons with diabetes and age-standardized based on the 2000 U.S. standard population.

^†^ Alaska, Vermont, and Wyoming were excluded because of the small annual number (<50) of new ESRD-D cases during the study period.

^§^ Rates declined from 2000 to 2011 (APC = -6.0% per year [95% CI: -7.1% to -4.9%], p<0.001), and then leveled off from 2011 to 2014 (APC = 5.2% per year [-1.4% to 12.2%], p = 0.11).

^¶^ Rates declined from 2000 to 2012 (APC = -5.6% per year [95% CI: -7.4% to -3.8%], p<0.001), and then leveled off from 2012 to 2014 (APC = 8.4% per year [-11.7% to 33.0%), p = 0.40).

**FIGURE 2:**
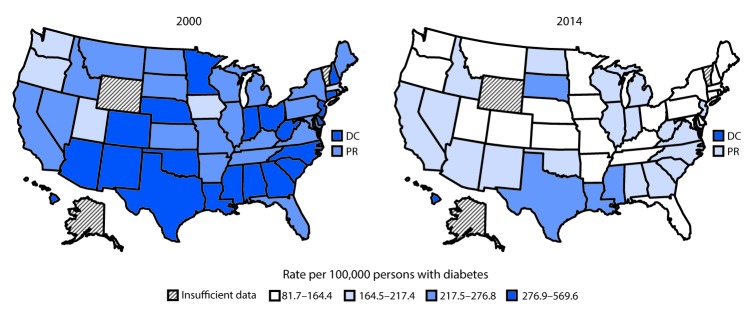
Age-standardized incidence* of end-stage renal disease attributed to diabetes (ESRD-D) among adults aged ≥18 years with diagnosed diabetes, by state^†^ — U.S. states, the District of Columbia, and Puerto Rico, 2000 and 2014^§^ **Abbreviations:** DC = District of Columbia; PR = Puerto Rico. * Rate per 100,000 persons with diabetes and age-standardized based on the 2000 U.S. standard population. ^†^ Alaska, Vermont, and Wyoming were excluded because of the small annual number (<50) of new ESRD-D cases. ^§^ Legend categories were created using ranks based on the combined 2000 and 2014 rates.

## Discussion

ESRD is a costly and disabling condition that often results in premature death (*1*). During 2000–2014, the overall age-standardized incidence of ESRD-D among adults with diagnosed diabetes decreased by 33%. Rates declined significantly in most states, DC, and Puerto Rico. In 2014, the highest rates were in DC and Hawaii. Continued awareness and interventions to reduce the prevalence of risk factors for kidney failure, improve diabetes care, and reduce the incidence of type 2 diabetes might sustain these positive trends.

The 33% decline in ESRD-D incidence from 2000 to 2014 reported here is similar to the 28% decline reported using 2000–2010 nationally representative surveillance data (*3*). Reasons for the decline in ESRD-D incidence cannot be determined from surveillance data. However, reasons for the decline might include reductions in risk factors for kidney failure (e.g., hyperglycemia and hypertension) in the diabetic population or better treatment of kidney disease, including the use of angiotensin-converting enzyme inhibitors or angiotensin-receptor blockers, which slow the decline in kidney function in addition to lowering blood pressure, thus delaying the onset of ESRD-D (*6*).

Although ESRD-D incidence rates are declining, the number of patients with newly diagnosed ESRD-D is likely to increase as the number of persons with diabetes increases (*2*). Furthermore, one in three adults with diabetes is estimated to have chronic kidney disease (i.e., kidney damage or reduced kidney function); however, most persons with chronic kidney disease are unaware that they have it (*7*). Early detection and better management of chronic kidney disease in persons with diabetes can slow its progression to ESRD, prevent complications, and improve cardiovascular outcomes (*7*). Testing for urine albumin, which is an early marker of kidney disease, is recommended for all patients with diabetes, and treatment with angiotensin-converting enzyme inhibitors or angiotensin-receptor blockers is indicated for persons with diabetes and hypertension (*8*). Effective interventions to improve blood glucose levels and blood pressure control might prevent or delay the onset of kidney disease (*7*) in adults with diabetes. To support primary prevention, effective community-based approaches to prevent obesity and increase physical activity, along with type 2 diabetes prevention programs targeted to populations at high risk, might reduce the incidence of type 2 diabetes, and consequently, diabetic kidney disease (*9*).

The findings in this report are subject to at least four limitations. First, data on ESRD treatment were based on reports to CMS; patients whose treatment was not reported to CMS (e.g., persons who refused treatment or who died from ESRD before receiving treatment) were not included and might result in an underestimation of incidence. Second, revised diagnostic criteria for diabetes in 1997 might have led to the detection of more persons with diabetes earlier in the disease process (persons who have not had diabetes long enough to develop ESRD-D) (*8*) and might result in an underestimation of incidence. Third, the estimated population with diagnosed diabetes was based on self-reports. Although self-report of diabetes is highly accurate (persons with diagnosed diabetes are likely to report having diabetes) (*10*), the total number of adults with diabetes is underestimated, which thus results in an overestimation of ESRD-D incidence. Finally, BRFSS survey methods changed in 2011 potentially confounding interpretation of trends. However, using different surveillance data to estimate the U.S. diabetic population yielded a similar overall decline in ESRD-D incidence rates (*3*).

CDC works with public and private partners to reduce the incidence of type 2 diabetes and to improve outcomes for persons with diabetes. In 2013, CDC assisted state health departments in implementing diabetes self-management education and training programs and strategies to increase use of diabetes self-management education and training by persons with diabetes. Diabetes self-management education and training is an important component of integrated diabetes care, teaching patients about diabetes and strategies they can use to manage their disease. CDC's National Diabetes Prevention Program (https://www.cdc.gov/diabetes/prevention) supports the nationwide implementation of evidence-based, structured lifestyle programs to prevent or delay the onset of type 2 diabetes among persons with prediabetes (persons who have blood glucose levels that are elevated, but not high enough to be diagnosed as diabetes). CDC's U.S. Diabetes Surveillance System (https://www.cdc.gov/diabetes/data) monitors diabetes and its risk factors and complications, including ESRD-D, to assess progress in diabetes prevention and control (*2*). CDC’s Chronic Kidney Disease Surveillance System (https://www.cdc.gov/ckd/surveillance) monitors the prevalence of chronic kidney disease (i.e., before ESRD) and its risk factors in the U.S. population and tracks progress in chronic kidney disease prevention, management, and control.

**Summary**

**What is already known about this topic?**

The incidence of end-stage renal disease attributed to diabetes (ESRD-D) in the U.S. population with diagnosed diabetes began to decline in the mid-1990s.

**What is added by this report?**

During 2000–2014, the age-standardized incidence of ESRD-D has continued to decline significantly in the United States and in most states, the District of Columbia, and Puerto Rico. No state experienced an increase in rates.

**What are the implications for public health practice?**

Continued awareness of diabetes and interventions to reduce the prevalence of risk factors for kidney failure, improve diabetes care, and prevent type 2 diabetes might sustain the decline in ESRD-D incidence rates in the population with diagnosed diabetes.

## Footnotes

*
https://www.cdc.gov/brfss/annual_data/2014/pdf/2014_dqr.pdf .

## References

[1] United States Renal Data System (USRDS). 2017 USRDS annual data report: epidemiology of kidney disease in the United States. Bethesda, MD: US Department of Health and Human Services, National Institutes of Health, National Institute of Diabetes and Digestive and Kidney Diseases; 2016. https://www.usrds.org/adr.aspx

[2] CDC. U.S. Diabetes Surveillance System. Atlanta, GA: US Department of Health and Human Services, CDC; 2017. https://gis.cdc.gov/grasp/diabetes/DiabetesAtlas.html

[3] Gregg EW, Li Y, Wang J, Changes in diabetes-related complications in the United States, 1990–2010. N Engl J Med 2014;370:1514–23. 10.1056/NEJMoa1310799 24738668

[4] CDC. Surveillance Resource Center: methodologic changes in the Behavioral Risk Factor Surveillance System in 2011 and potential effects on prevalence estimates. Atlanta, GA: US Department of Health and Human Services, CDC; 2013. https://www.cdc.gov/surveillancepractice/reports/brfss/brfss.html

[5] Kim HJ, Fay MP, Feuer EJ, Midthune DN. Permutation tests for joinpoint regression with applications to cancer rates. Stat Med 2000;19:335–51. 10.1002/(SICI)1097-0258(20000215)19:3<335::AID-SIM336>3.0.CO;2-Z 10649300

[6] Afkarian M, Zelnick LR, Hall YN, Clinical manifestations of kidney disease among US adults with diabetes, 1988–2014. JAMA 2016;316:602–10. 10.1001/jama.2016.10924 27532915 PMC5444809

[7] CDC. National chronic kidney disease fact sheet, 2017. Atlanta, GA: US Department of Health and Human Services, CDC; 2017. https://www.cdc.gov/diabetes/pubs/pdf/kidney_factsheet.pdf

[8] American Diabetes Association. Standard of medical care in diabetes—2017. Diabetes Care 2017;40(Suppl 1):S75–98.27979896

[9] Albright AL, Gregg EW; National Diabetes Prevention Program. Preventing type 2 diabetes in communities across the U.S.: the National Diabetes Prevention Program. Am J Prev Med 2013;44(Suppl 4):S346–51. 10.1016/j.amepre.2012.12.009 23498297 PMC4539613

[10] Saydah SH, Geiss LS, Tierney E, Benjamin SM, Engelgau M, Brancati F. Review of the performance of methods to identify diabetes cases among vital statistics, administrative, and survey data. Ann Epidemiol 2004;14:507–16. 10.1016/j.annepidem.2003.09.016 15301787

